# Novel Ordered Stepped-Wedge Cluster Trial Designs for Detecting Ebola Vaccine Efficacy Using a Spatially Structured Mathematical Model

**DOI:** 10.1371/journal.pntd.0004866

**Published:** 2016-08-10

**Authors:** Ibrahim Diakite, Eric Q. Mooring, Gustavo E. Velásquez, Megan B. Murray

**Affiliations:** 1 Department of Global Health and Social Medicine, Harvard Medical School, Boston, Massachusetts, United States of America; 2 Department of Epidemiology, Harvard T.H. Chan School of Public Health, Boston, Massachusetts, United States of America; 3 Division of Infectious Diseases, Brigham and Women’s Hospital, Boston, Massachusetts, United States of America; 4 Division of Infectious Diseases, Massachusetts General Hospital, Boston, Massachusetts, United States of America; 5 Department of Medicine, Harvard Medical School, Boston, Massachusetts, United States of America; 6 Division of Global Health Equity, Brigham and Women’s Hospital, Boston, Massachusetts, United States of America; 7 Partners In Health, Boston, Massachusetts, United States of America; Oswaldo Cruz Foundation, BRAZIL

## Abstract

**Background:**

During the 2014 Ebola virus disease (EVD) outbreak, policy-makers were confronted with difficult decisions on how best to test the efficacy of EVD vaccines. On one hand, many were reluctant to withhold a vaccine that might prevent a fatal disease from study participants randomized to a control arm. On the other, regulatory bodies called for rigorous placebo-controlled trials to permit direct measurement of vaccine efficacy prior to approval of the products. A stepped-wedge cluster study (SWCT) was proposed as an alternative to a more traditional randomized controlled vaccine trial to address these concerns. Here, we propose novel “ordered stepped-wedge cluster trial” (OSWCT) designs to further mitigate tradeoffs between ethical concerns, logistics, and statistical rigor.

**Methodology/Principal Findings:**

We constructed a spatially structured mathematical model of the EVD outbreak in Sierra Leone. We used the output of this model to simulate and compare a series of stepped-wedge cluster vaccine studies. Our model reproduced the observed order of first case occurrence within districts of Sierra Leone. Depending on the infection risk within the trial population and the trial start dates, the statistical power to detect a vaccine efficacy of 90% varied from 14% to 32% for standard SWCT, and from 67% to 91% for OSWCTs for an alpha error of 5%. The model’s projection of first case occurrence was robust to changes in disease natural history parameters.

**Conclusions/Significance:**

Ordering clusters in a step-wedge trial based on the cluster’s underlying risk of infection as predicted by a spatial model can increase the statistical power of a SWCT. In the event of another hemorrhagic fever outbreak, implementation of our proposed OSWCT designs could improve statistical power when a step-wedge study is desirable based on either ethical concerns or logistical constraints.

## Author Summary

When a vaccine is developed, it undergoes a series of tests to assess its safety and effectiveness. The last of these is called a Phase III clinical trial, in which the vaccine is tested on a subset of the population before it is approved for general use. A randomized controlled trial (RCT) in which individuals are randomized to receive vaccine or placebo is the most direct and efficient trial design to assess the efficacy of a vaccine. However, in circumstances where a disease has a very high mortality rate (such as Ebola virus disease), the use of placebo is ethically questionable, especially when there is strong evidence that a vaccine will be safe and efficacious. Vaccine trials often must also address logistical constraints that prevent the introduction of the vaccine to the entire trial population in certain resource-poor settings. These issues were front and center in discussions about vaccine trials during the 2014 Ebola outbreak. The medical community faced questions on the clinical trial design that best balanced tradeoffs between ethical concerns, logistics, and statistical rigor. In this study, we propose and assess novel “ordered stepped-wedge cluster trial” designs as an alternative to mitigate these tradeoffs.

## Introduction

The 2014 Ebola virus disease (EVD) epidemic is the largest recorded outbreak of any filovirus infection, primarily affecting three major countries in West Africa: Guinea, Liberia, and Sierra Leone. The three countries combined had a total of 15,901 cases (confirmed, probable and suspected) and 5,674 deaths as of November 26, 2014, when the epidemic peaked in the affected regions [1]. At that time, many candidate vaccines were proposed for Phase III trials in the affected countries, with different vaccine trial designs suggested for each region. Between April 1, 2015, and July 20, 2015, a Phase III trial in Guinea assessed the efficacy of a Zaire Ebolavirus vaccine (rVSV-ZEBOV) [2]. The design was a ring vaccination cluster-randomized trial, where the trial population was made up of clusters of all contacts and contacts of contacts of laboratory-confirmed Ebola cases. Thus, a robust contact tracing system was an essential component of the trial. Unfortunately, the 2014 Ebola outbreak has demonstrated that it requires valuable time to establish a reliable contact tracing system in the setting of damaged public health infrastructure, a severe shortage of health care workers, and community resistance, amongst other reasons [3].

The stepped-wedge cluster trial (SWCT) was another trial design proposed to test a candidate vaccine in Sierra Leone (SL) during the outbreak. In contrast with ring vaccination, the SWCT does not rely on a contact tracing system [4,5]. The trial population is made of geographically distinct clusters that are randomly and sequentially assigned to vaccination. This design is desirable when vaccination cannot be introduced to all clusters at once due to logistical or financial reasons, and has the ethical advantage of not intentionally withholding vaccines from unvaccinated clusters while they serve as control groups. However, when the vaccine is expected to be efficacious, the risk of infection is predicted to vary between clusters over time, and these different risks can be predicted, randomly choosing a cluster to be treated fails to prioritize those at highest risk (which undermines the ethical advantage of the SWCT). Furthermore, Bellan et al. [6] have shown that spatiotemporal variation in infection risk undermines the statistical power of the SWCT. Here, we propose novel “ordered stepped-wedge cluster trial” (OSWCT) designs to address these limitations of the standard SWCT.

OSWCT designs differ from SWCT when clusters are predicted to have different infection risks. Specifically, OSWCT designs use conditional randomization to assign clusters to vaccination. The cluster to vaccinate at a given time point is randomly selected from a subset of clusters that are likely to have a higher infection risk. We considered three strategies to identify the highest risk clusters: based on the order of first EVD case occurrence in each cluster as projected by a spatial model, based on the observed highest incident cases two weeks previously, or the highest projected weekly incidence. By prioritizing clusters, OSWCT mitigates the ethical dilemma of randomly assigning treatment to clusters when they are predicted to have low infection risk.

In this study we assessed the statistical power of these novel trial designs. We simulated and estimated the statistical power of all the designs in the following steps. First, we constructed a metapopulation model that combines EVD transmission and individuals’ movements between regions in order to predict the spatiotemporal trends of the disease. Second, we used either the observed or modeled incidence data within districts of SL to assign clusters to receive vaccination for the OSWCT designs. Third, we used a stochastic model to simulate all trial designs, and finally we used a nonparametric method (permutation test) to analyze the simulated data and to estimate the statistical power of trial designs.

## Methods

### Data sources

#### Case count data

The World Health Organization (WHO) provided weekly updated data on the number of newly confirmed and probable Ebola cases for each district in Sierra Leone starting from the week of May 19, 2014, and for each county in Liberia starting the week of March 17, 2014. These were derived from two sources: a daily situation report from the Ministries of Health (MOH) [7] which contained a summary of the total number of probable and laboratory confirmed EVD cases in each country; and an individual-level patient database [8] or “linelist” which contained information on the symptoms, diagnosis, and outcomes for each probable or confirmed Ebola case. WHO defined a “probable case” as a person who a clinician suspected of having Ebola, or who died from “suspected” Ebola and had an epidemiological link to a confirmed case but was not tested for the disease. A probable or suspected case was reclassified as confirmed if that person tested positive for Ebola by a PCR-based test. WHO continuously updated the patient database as a result of the ongoing reclassification, retrospective investigations, and availability of laboratory results.

#### Geospatial data

**Fig 1:**
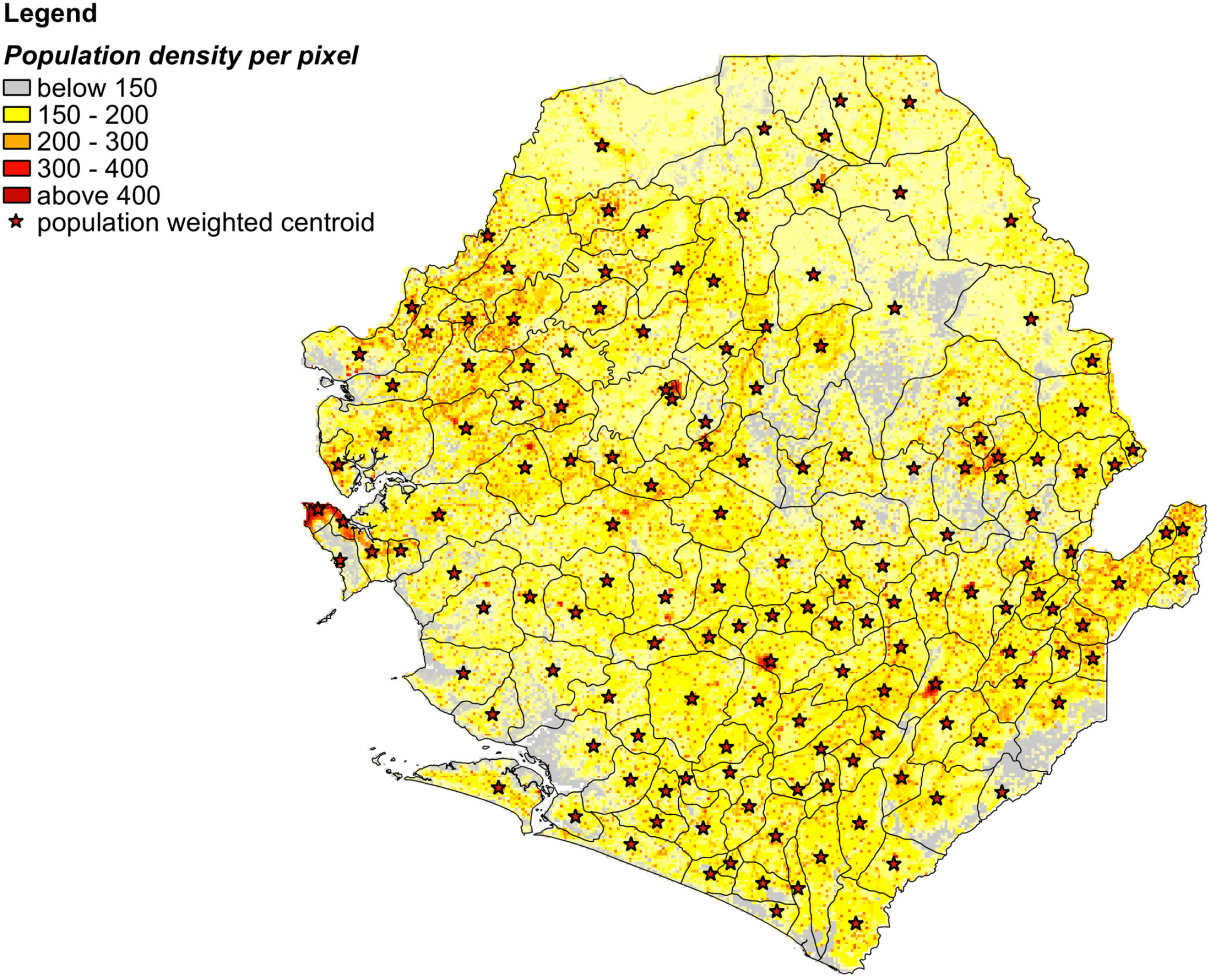
The chiefdoms of Sierra Leone with their population-weighted centroids.

We used the Database of Global Administrative Areas (GADM) [9], which provides data on the location of the world's administrative areas, to obtain the boundaries of the 153 chiefdoms within the 14 districts of SL. We estimated the population density for each chiefdom in SL from the Oak Ridge National Laboratory’s global population distribution data LandScan [10]; the LandScan algorithm uses spatial data and imagery analysis technologies to disaggregate census counts within an administrative boundary. To calculate the distance between chiefdoms, we identified population-weighted centroids for each using the geographic information system software ArcGIS version 10.2 and then measured the pairwise distance between centroids (Fig 1).

### Model specification

#### EVD transmission model

**Fig 2:**
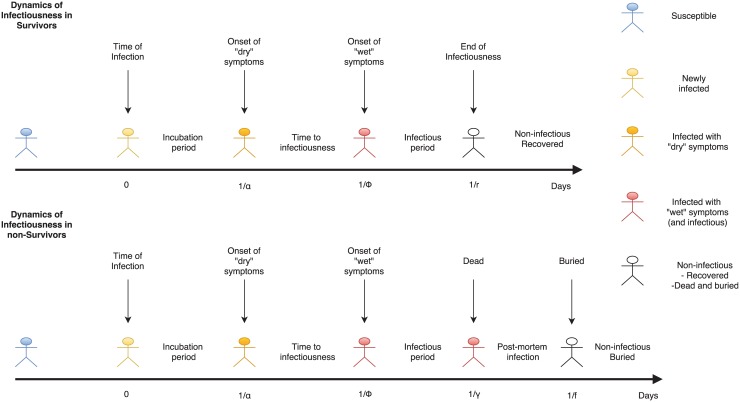
Timeline of EVD at the individual level.

We modified a previously published SEIR model [11] to capture the dynamics of EVD transmission in the 2014 SL and Liberian epidemic. Here, we assumed that susceptible individuals [S] enter the exposed [E] compartment at a rate (*β*) that reflects the frequency of close contact with body fluids of infectious people [I] and the probability of an infection event after contact. We assume that *β* varies depending on the status of infectious cases, and that transmission rates from patients in Ebola treatment units (ETUs) (*β*^*etu*^) are lower than those from patients in the community (*β*^*c*^), which are again lower than those from patients who have died and are undergoing burial (*β*^*f*^). After an incubation period (1/α), infected individuals develop “dry” symptoms (fever, myalgia, headache, presence of oropharyngeal lesions, nausea, abdominal pain, and rash); these can prompt clinical diagnosis, identification, and isolation of an EVD case [Fe] but they occur prior to the onset of infectiousness which occurs when the infected person develops “wet” symptoms (vomiting, diarrhea, coughing, hemorrhage) (1/) days after the onset of “dry” symptoms [12,13]. We assumed that infectious individuals [I] can either recover at rate (r) or die at rate (δ). Fig 2 shows a timeline of Ebola infection and the relevant rates.

**Fig 3:**
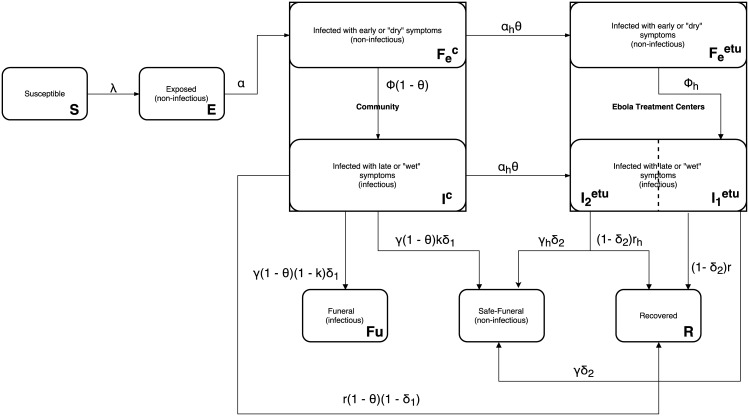
Natural history of Ebola virus disease at the population level.

Fig 3 summarizes our assumptions regarding the clinical management of patients with EVD. We assumed that patients with dry and wet symptoms were admitted to ETUs with probability (θ) and that the remainder remained in the community. We also assumed that EVD patients who died in ETUs received “safe” burials (i.e., were handled in a way to prevent exposure to body fluids) while a proportion (1-k) of those who died in community received “unsafe” burials and remained infectious for the duration of traditional funerals, (1/f) days (see full equations in the S1 Appendix).

#### Gravity model

We linked the EVD transmission model to a spatial model in order to capture the geographic and temporal trends due to human mobility between regions of SL. Here, we used a gravity model, which assumes that mobility between two regions is directly proportional to their population sizes and inversely proportional to the distance between them:

$$m\left(i,j\right)=\rho \;\frac{P\left(i\right).P\left(j\right)}{D{\left(i,j\right)}^{n}},\;\;\;\;i,j=1,\ldots ,T_{r}$$ (1)

where *P*(*i*) is the population in region *i*, *D*(*i*, *j*) is the distance between the population weighted centroids of regions *i* and *j*; and *T*_*r*_ is the total number of regions. *ρ* is the proportionality factor, and *n* a constant that determines the strength of the dependence of mobility rate on distance. Here we refer to the combined transmission model and gravity model as the metapopulation model.

### Model parameterization

#### Static parameters

We obtained parameters for the incubation period, the time from onset of dry symptoms to wet symptoms, the duration of infectiousness, and the time from onset of wet symptoms to death from our previous systematic review [14]. We estimated the time from onset of any symptoms to hospitalization (1/*α*_*h*_) from a published case series [15].

#### Time-varying parameters

To capture the impact of public health interventions on the Ebola epidemic, we assumed that the major international response to Ebola that began in September 2014 altered the following: the probability that an EVD case was admitted to an ETU (θ), the case fatality ratio (δ), and the proportion (k) of EVD deaths within the community that were followed by a safe burial. We assumed that the probability of admission to an ETU was a function of the number of ETU beds available and we modeled this based on the bed count reported by the Humanitarian Data Exchange [16] for the period from September 16, 2014, to January 19, 2015. We found that the case fatality ratio [1] in SL for the months of September through December 2014 was best fit to a cubic regression curve. Lastly, the time-varying probability that community deaths received safe burials was estimated from WHO situation reports [1].

### Model calibration

#### Initial dynamics of infection

We calibrated the EVD transmission model in SL to the cumulative number of Ebola cases from May 19, 2014, to September 16, 2014, by fitting transmission coefficients and the probability of hospitalization to minimize the squared differences between modeled and reported disease counts.

#### Calibration of the gravity model

Because the daily and weekly EVD cases [7,8] were only reported at the district or country level in SL as opposed to at the chiefdom level, with the best-fit parameter values obtained from the initial transmission model we next coupled the transmission model to the gravity model to fit the values of ρ and n that best reproduced the reported order and timing of the initial case in each district.

#### Changing dynamics of infection over time

The onset of the international response to Ebola in West Africa in September involved the establishment and staffing of ETUs, the introduction of personal protective equipment for health care workers (HCWs), the dissemination of information about transmission, and the implementation of intermittent social distancing measures. Using incidence data from September 16, 2014, to January 19, 2015, we fit post-intervention transmission coefficients. However, the international response was implemented gradually, so we also fit exponential decay parameters to represent the progressive transition from pre-intervention transmission rates to post-intervention transmission rates.

### Model validation

We tested the validity of the metapopulation model by comparing the model output to the epidemic trajectory in Liberia, assuming that the pre-intervention transmission model and human mobility parameters for EVD were the same as those in SL. We evaluated goodness of ordering and timing with the Spearman correlation coefficients for the observed versus expected order and timing of first case occurrence in each county.

We also tested the extent to which the metapopulation model’s ordering of first case occurrence depended on the disease parameters used in the EVD transmission model. We therefore simulated a hypothetical outbreak of smallpox, varicella (chickenpox), and measles in the same regions of SL, with model parameter values drawn from the published literature (see S1 Appendix). We then coupled each of the new transmission models to the gravity model and reran the metapopulation model to obtain new ordering of first case occurrence. We measured the correlation between the metapopulation model’s ordering of the Ebola outbreak versus the hypothetical smallpox, varicella, or measles outbreaks by evaluating Spearman correlation coefficients.

### Vaccine trial design

**Fig 4:**
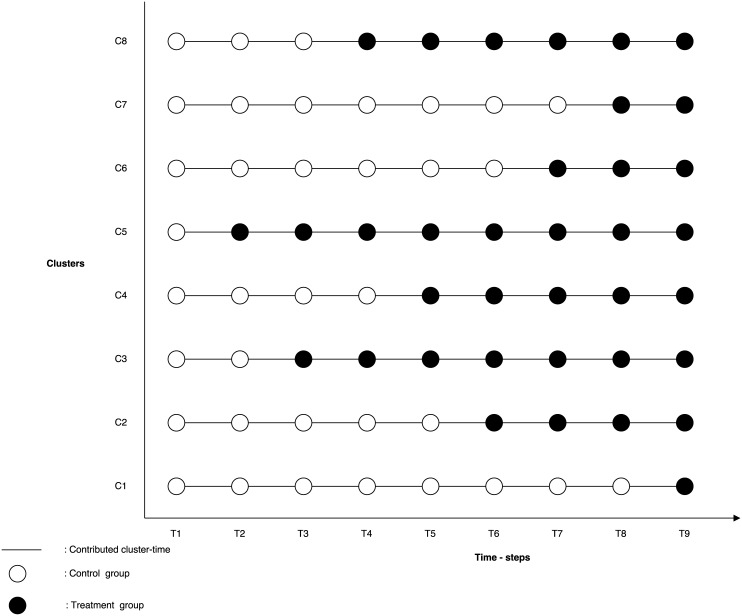
Traditional SWCT design. All clusters contribute cluster time from the beginning to the end of the study, and clusters are randomly assigned to treatment. Here we only show 8 of the 14 clusters.

We first considered a standard stepped-wedge cluster trial (SWCT) [4,5] in which a single new cluster is randomly selected to receive vaccination at each pre-allocated time point during the trial period (Fig 4). In all of our simulations, a new cluster was vaccinated each week. Each cluster was considered part of the control group until it crossed over to the treatment arm of the trial, and all clusters were followed from the beginning of the trial until it ended. Vaccine efficacy was estimated by comparing incidence in the vaccinated and unvaccinated clusters at each time step [17].

**Fig 5:**
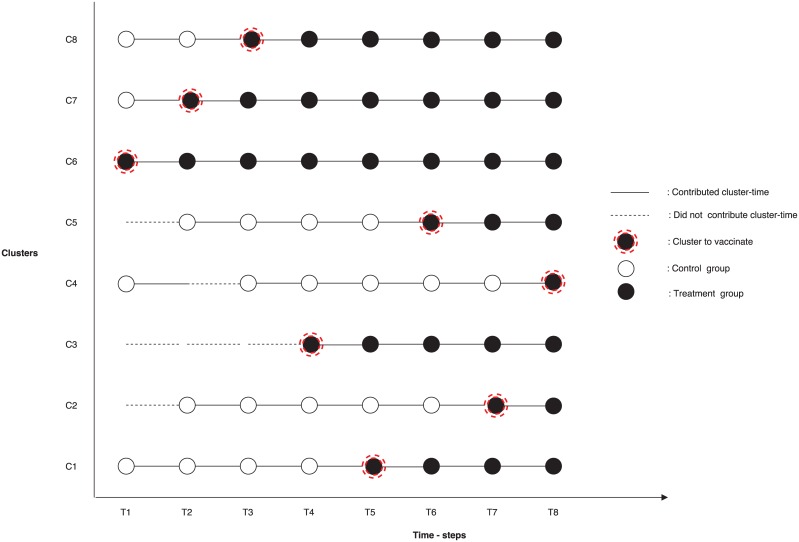
OSWCT designs. A cluster contributes cluster-time (solid line) in time [t, t+1) if it is selected among the top N highest risk clusters or if it has been previously vaccinated, otherwise it does not contribute (dash line) cluster time in that time interval. White dots represent selected clusters that serve as controls, black dots serve as the treatment group, and the black dot with dashed circle represents the cluster that was randomly selected among the top N highest risks clusters to receive treatment at that time point. Here we only show 8 of the 14 clusters.

While SWCTs are often used to evaluate the impact of interventions for chronic noncommunicable diseases, they encounter methodological problems when they are used to evaluate those for infectious diseases. Specifically, the force of infection over the course of an epidemic often varies widely between clusters and changes over time. To account for such variation between clusters, we proposed “ordered stepped-wedge cluster trial” (OSWCT) designs in which clusters are assigned to a treatment group based either on the observed incidence data or on a projection of cases derived from a spatially structured transmission model (Fig 5). The overarching purpose of this design is to randomly select for intervention a cluster from a more homogeneous subset of clusters.

We simulated and assessed the statistical power of four vaccine study designs: a standard SWCT and three types of novel OSWCTs, one in which we ordered clusters according to the districts with the most observed cases in the two weeks prior (data-OSWCT), a second in which we ordered clusters based on the ordering of first case occurrence in districts by our spatially structured EVD model (first-OSWCT), and a final design in which we ordered clusters based on the highest predicted incidence at each implementation time step (peak-OSWCT). For all OSWCTs, we randomly selected the cluster to be vaccinated at each time step from among the top N (e.g., 4 or 5) unvaccinated clusters ranked by the ordering strategy, and the remaining N-1 unvaccinated clusters served as the control group (Fig 5). Once vaccinated, clusters continued to contribute cluster time until the end of the trial. When there were N or fewer unvaccinated clusters remaining, OSWCT designs became analogous to the standard SWCT design.

We chose these different ordering strategies because the first-OSWCT design does not rely on availability of epidemic data to be able to order clusters, therefore it can be used when one is planning an OSWCT early in the outbreak before all the regions have observed their first case. However, if a trial does not start until later in the outbreak after the disease has spread to all regions, then ordering based on first case occurrence is no longer an efficient strategy. Ordering strategies based on the highest observed incidence two weeks prior (data-OSWCT) or on a projection of weekly highest cases (peak-OSWCT) are applicable to trials starting early or late in the outbreak. In principle, a peak-OSWCT could be designed using a metapopulation model informed only by baseline geographic data and initial estimates of model parameters. However, a model that takes into account surveillance data up to the start of the trial as well as data on the implementation of other disease control interventions is likely to have greater predictive ability. Another advantage of the transmission model-driven ordering schemes (first-OSWCT and peak-OSWCT) is that the randomization can be undertaken before any cluster receives vaccination, compared to the data-driven ordering scheme (data-OSWCT) which relies on the availability of surveillance data and thus requires the randomization be updated as the trial progresses.

### Vaccine trial simulations

Following Bellan et al. [6], we used a stochastic model to simulate vaccine trials. We assumed that the trial would be conducted in geographically distinct clusters, with each drawn from the population of one of the 14 districts of Sierra Leone. In April 2015, the U.S. Centers for Disease Control and Prevention (CDC) partnered with various institutions in SL including its Ministry of Health and Sanitation (MOHS) to conduct a Phase II and Phase III clinical trial named Sierra Leone Trial to Introduce a Vaccine against Ebola (STRIVE) [18,19]. The study enrolled HCWs and other frontline workers to assess the safety and efficacy of an Ebola vaccine candidate. Participants were randomized to receive vaccination immediately (on the day of enrollment or within seven days) or to receive delayed vaccination (about six months later).

We consequently assumed our simulated trials to be conducted in high-risk individuals, such as HCWs and burial team members, and that all the simulated clusters would be of equal size. We assumed that without effective vaccination, a proportion (*p*) of the reported incident cases in the district would occur in the corresponding cluster; using proportionality constant (*p*), we derived the cluster-level hazard (*H*_*p*_) to be directly proportional to the incidence of cases in the corresponding district. The force of infection risk for each individual within a specific cluster and time interval is *H*_*P*_ * *ε*, where *ε* captures expected variation in individual infection that is log-normally distributed with mean 1 and standard deviation 1. We assumed that a cluster could be fully vaccinated within one week and that the vaccine effect began after a delay of (*di*).

STRIVE was expected to enroll 6,000 participants [19]. We assumed that each of the 14 simulated clusters had 430 individuals in order to arrive at a simulated trial size of 6020 individuals, to approximately match the originally expected sample size of the STRIVE trial. In our baseline simulation, we assumed that a hypothetical study began early during the outbreak before all districts were reported to have cases (between mid-May to late August 2014). We assumed that 5.2% of the total number of cases in each district would have occurred within high-risk groups, consistent with CDC reports [20]. Based on the preliminary results of the rVSV ring vaccination trial in Guinea [2], we assumed that vaccine efficacy (*ve*) was 90% and that the time from vaccination to the onset of vaccine-induced immunity (*di*) was one week. In sensitivity analyses, we varied trial start dates as well as values (*p*), (*ve*) and (*di*). When we simulated a trial that started late during the course of the outbreak (between late November 2014 to mid-March 2016), we dropped the ordering design based on first case occurrence in districts (first-OSWCT), since all districts were predicted to have their first EVD case by late August 2014.

### Statistical analysis

We used a nonparametric method (permutation test) described by Bellan et al. [6] to analyze the simulated data for all designs including SCWT as well as all types of OSWCT. For each cluster i during each week t, we calculated the simulated number of infected individuals (Y_it_), their vaccine status (X_it_), their cluster-time status (CT_it_) (that is, an indicator variable set to 1 for previously vaccinated clusters and the set of high-ranking clusters from which the vaccinated cluster was randomly drawn at time t), and the vaccinated and unvaccinated person-time (PY_it_) for all trial participants. We analyzed the data with a generalized estimating equation (GEE), log(E(Y_it_)) = C_i_ + β_vac_X_it_ + β_ct_ CT_it_ + β_time_ t + log(PY_it_), where C_i_ is a cluster-level random effect, and we estimated β_vac_, the log relative hazard of infection among vaccinated compared to the unvaccinated. We computed the estimated vaccine efficacy as $\hat{\text{V}}\text{e}=1\;–\text{ exp }({\hat{\beta }}_{\text{vac}})$. We also computed the magnitude of bias in the vaccine efficacy estimate as $\hat{\text{V}}\text{e}-\text{Ve}$.

Under the null hypothesis of no vaccine effect, the time at which a cluster received vaccination will have no impact on the number of cases that occur. We therefore permuted 1000 times the order in which clusters were vaccinated, keeping individuals’ final infection status unchanged, and re-estimated β_vac_ for each permutation. We calculated a Wald statistic [21] for each permuted data set and tested the null hypothesis of no vaccine effect with a two-sided significance level α = 0.05. To estimate the power to detect vaccine efficacy, we repeated this process 2000 times.

## Results

### Model parameterization and calibration

**Fig 6:**
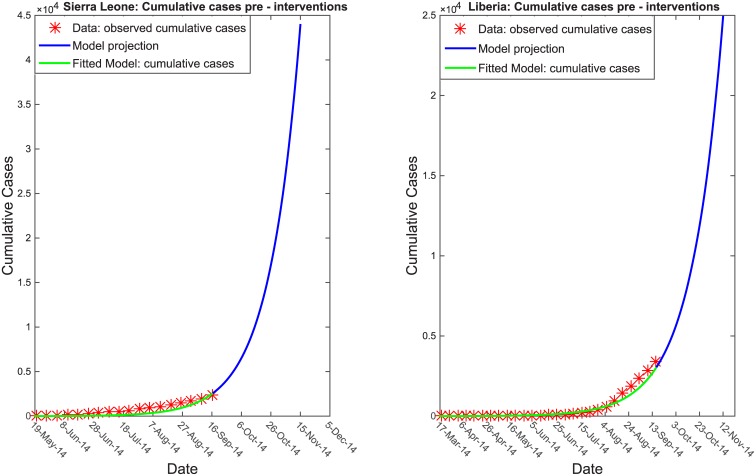
Initial dynamics of infection. Transmission model calibrated to the cumulative number of Ebola cases reported in SL from May 19, 2014, to September 16, 2104, and in Liberia from March 17, 2014, to September 16, 2014.

Table 1 gives the set of parameters that best fit the reported case counts for Sierra Leone in both the pre and post-intervention periods. Prior to the scale up of intervention measures we found the transmission coefficients of 0.48 in the community, 0.16 in ETUs, and 0.54 in funerals. After the implementation of intervention controls into the model, we estimated these transmission coefficients decreased by 81% in the community, 69% in ETUs and 52% at funerals. Fig 6 shows that the transmission model accurately fit the early disease trends reported in SL and Liberia through mid-September 2014. However, without any change in the transmission model parameters to reflect intervention measures, the model predicted an abrupt increase in cases.

**Table 1:** Metapopulation model parameters.

Parameter	Definition	Sierra Leone Values	Liberia Values	Reference
***EVD Natural History***				
α-1	Incubation period	7.34 days	-	[14]
ϕ^-1^	Time from onset of “dry” symptoms to onset of “wet” symptoms	6.05 days	-	[14]
α_h_^-1^	Time from onset of any symptoms to hospitalization	4 days	-	[15]
ϕ_h_^-1^ = ϕ^-1^ - α_h_^-1^	Time from hospitalization with “dry” symptoms to onset of “wet” symptoms	2.05 days	-	calculated
γ^-1^	Time from onset of “wet” symptoms to death	8 days	-	[14]
r^-1^	Duration of “wet” symptoms for survivors	9.40 days	-	[14]
r_h_^-1^ = r^-1^ - α_h_^-1^	Time from hospitalization with “wet” symptoms to end of “wet” symptoms for survivors	5.40 days	-	calculated
γ_h_^-1^	Time from hospitalization to death	4 days	-	[15]
f^-1^	Duration of traditional funerals	2 days	-	[11]
***Disease dynamic pre-intervention (up to 16 Sep 2014)***				
β_0_^c^	Transmission coefficient in the community	0.48 day^-1^	0.43 day^-1^	fitted
β_0_^etu^	Transmission coefficient in the ETUs	0.16 day^-1^	0.12 day^-1^	fitted
β_0_^f^	Transmission coefficient during funeral practices	0.54 day^-1^	0.60 day^-1^	fitted
θ	Probability of hospitalization	0.45	-	fitted
δ	Case fatality ratio	0.69	-	[22]
δ_1_ = $\frac{\delta r}{\delta r+\left(1-\delta \right)\;\gamma }$	Case fatality rate in the community	0.65	-	calculated
δ_2_ = $\frac{\delta \left(r+r_{h}\right)}{\delta \left(r+r_{h}\right)+\left(1-\delta \right)\left(\gamma +\gamma_{h}\right)}$	Case fatality rate in the ETUs	0.63	-	calculated
k	Proportion of decedents in community who receive safe burial	0	-	
***Disease dynamic post-intervention (16 Sep 2014–19 Jan 2015)***				
β_1_^c^	Final transmission coefficient in the community	0.09 day^-1^		fitted
β_1_^etu^	Final transmission coefficient in the ETUs	0.05 day^-1^		fitted
β_1_^f^	Final transmission coefficient during funeral practices	0.26 day^-1^		fitted
θ(t)	Probability of hospitalization	function of beds in ETUs		calibrated
δ	Case fatality ratio	cubic curve		calibrated
δ_1_	Case fatality rate in the community	function of δ(t)		†
δ_2_	Case fatality rate in the ETUs	function of δ(t)		†
k	Proportion of decedents in community who receive safe burial	linear increase		calibrated
q^c^	Exponential decay rate from β_0_^c^ to β_1_^c^ in the community	0.06		fitted
q^h^	Exponential decay rate from β_0_^etu^ to β_1_^etu^ in ETUs	0.55		fitted
q^f^	Exponential decay rate from β_0_^f^ to β_1_^f^ during funerals	19 x 10^−9^		fitted
***Gravity parameters***				
ρ	Gravity proportionality factor	6.31 x 10^−13^	-	fitted
n	Gravity constant	2.55	-	fitted

*Definition of abbreviations*: ETUs, Ebola treatment units; EVD, Ebola virus disease.

(-): Parameter values were assumed as in Sierra Leone.

^†^: Equations are the same as in the pre-intervention period.

**Fig 7:**
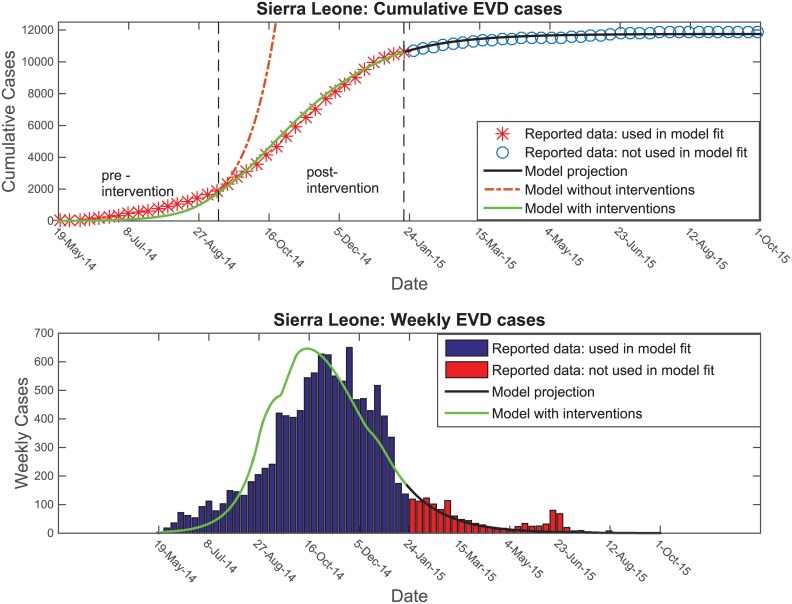
Changing dynamics of infection over time. Transmission model calibrated to the cumulative number of EVD cases reported in SL from May 19, 2014, to January 19, 2015 and comparison of observed and projected cases from January 19,2015 to October 1, 2015, (upper panel). The lower panel shows the model’s weekly projected cases overlaid on the reported weekly cases in SL.

**Fig 8:**
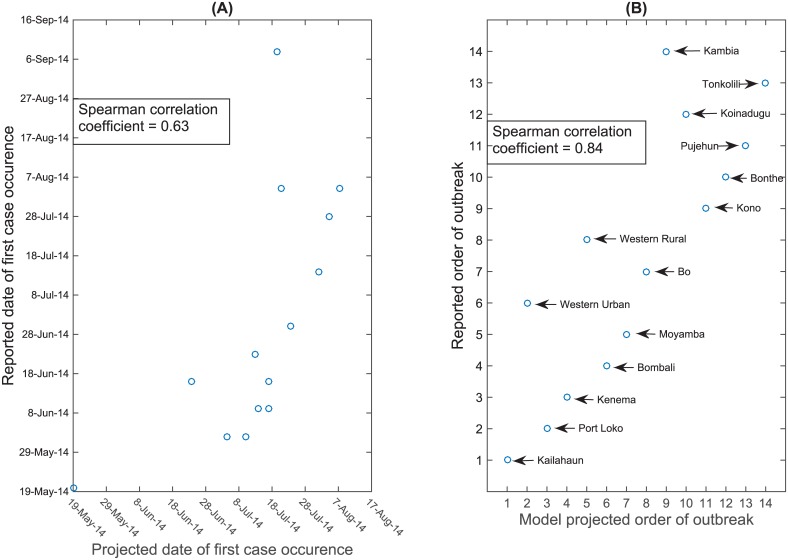
Order of first case occurrence in the districts of Sierra Leone. Correlation between spatial model projections and WHO reports on initial case dates (A) and ordering of outbreaks (B).

**Fig 9:**
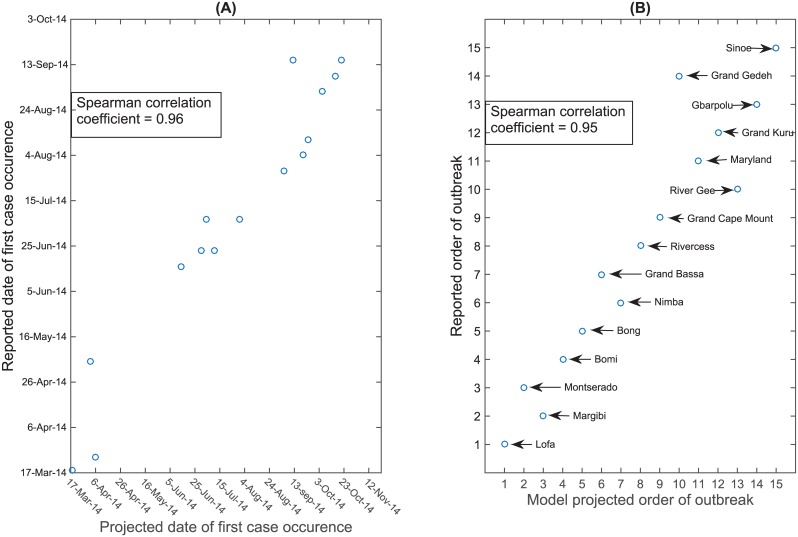
Order of first case occurrence in the counties of Liberia. Correlation between spatial model projections and WHO reports on initial case dates (A) and ordering of outbreaks (B).

To capture the impact of public health interventions on the Ebola epidemic after mid-September 2014, we changed the transmission model parameters including transmission parameters, the case fatalities rates, the probability of hospitalization, and the probability of safe burial. In Fig 7, we fit the transmission model with interventions to the reported data from the beginning of the outbreak up to mid-January 2015, whereas the modeled trajectory without intervention measures deviated from the reported data after mid-September 2014. The plot also shows a comparison of the model forecast with the reported data that we did not use for fitting the model from mid-January until October 2015. Fig 8 plots the projected order and timing of the first cases in each district of SL against the reported order; Spearman Correlation coefficients were 0.84 (*P* value <0.001) and 0.63 (*P* value <0.01), respectively. We obtained a similarly good fit for the projected order and timing of first county cases in Liberia with Spearman correlation coefficients of 0.95 (*P* value <0.001) and 0.96 (*P* value <0.001), respectively (Fig 9).

**Fig 10:**
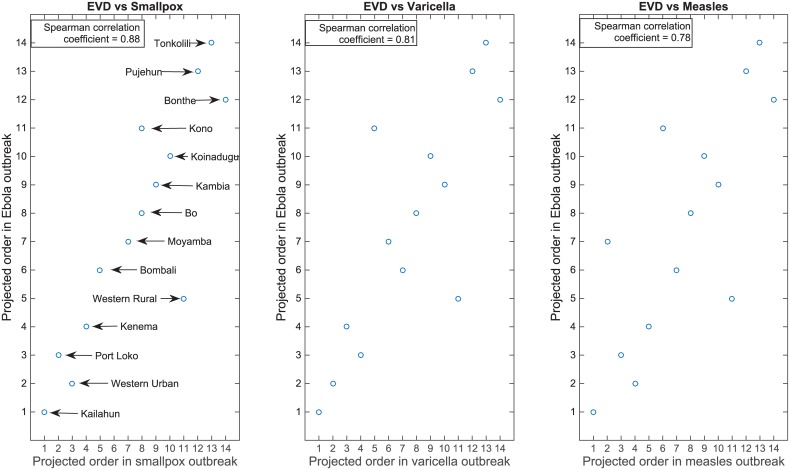
Order as function of transmission model. Comparison of the metapopulation model’s projection of first case occurrence within SL districts when parameters for EVD transmission are considered versus considering the parameters for smallpox, varicella, and measles transmission.

We found the metapopulation model’s projected ordering of first case occurrence within the districts to be consistent when we used different transmission model parameters for a hypothetical outbreak of smallpox, varicella, and measles in the same regions of SL. We obtained Spearman correlation coefficients of 0.88 (*P* value <0.001) between the order for EVD versus smallpox, of 0.81 (*P* value <0.001) for EVD versus varicella, and 0.78 (*P* value <0.001) for EVD versus measles (Fig 10).

### Statistical power for SWCT and OSWCT trial designs

**Fig 11:**
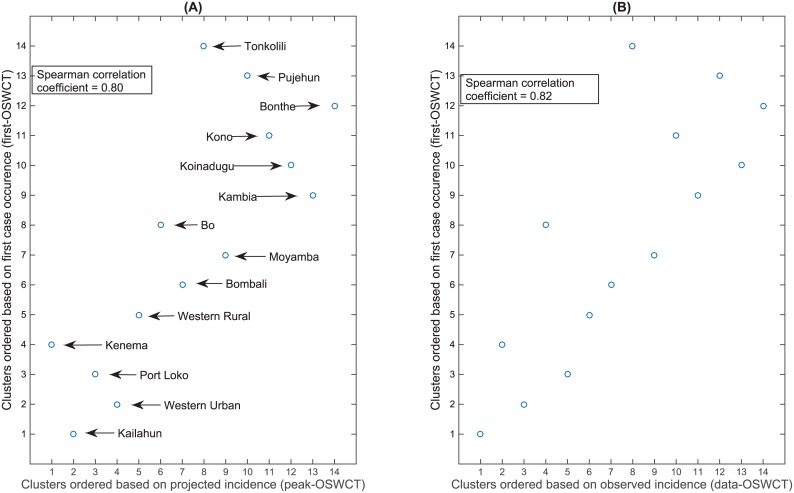
Comparison of clustering for the different ordering designs. (A) first-OSWCT vs. peak-OSWCT. (B) first-OSWCT vs. data-OSWCT. The trial was assumed to start 10 weeks after the onset of the outbreak.

We used the ordering of cases to simulate different vaccine trial designs in SL with clusters ordered to receive treatment based on random assignment (SWCT), on observed highest incidence in the two weeks prior (data-OSWCT), on the metapopulation model’s projected first case within districts (first-OSWCT), or on the highest weekly projected incidence (peak-OSWCT). For all of the OSWCT designs, the cluster to be vaccinated was randomly drawn from the 4 highest-ranked unvaccinated clusters (once 4 or fewer clusters remained, the ordered designs effectively operate as standard SWCTs with random assignment). When the trial started early during the outbreak (prior to September 2014) before all the districts observed their first case, we calculated the correlation between the different ways of ordering clusters, and we obtained similar correlation between first-OSWCT, data-OSWCT, and peak-OSWCT (Fig 11).

**Fig 12:**
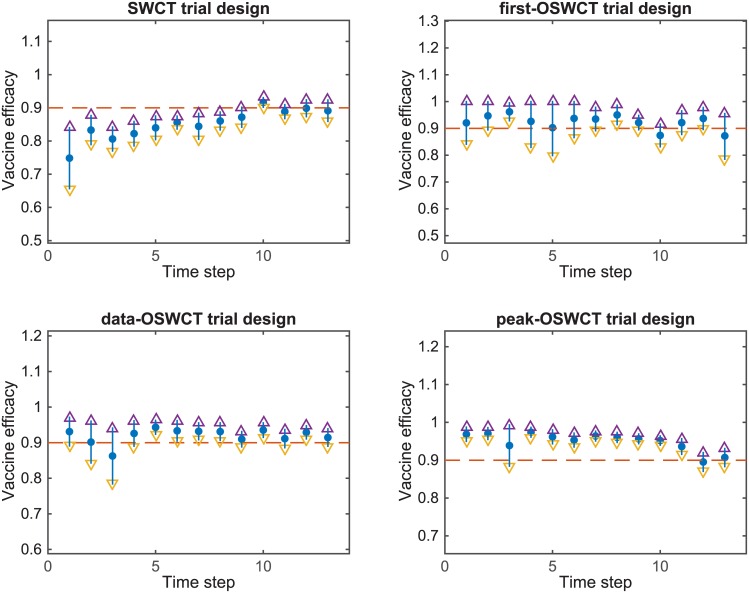
Measure of bias. Estimated vaccine efficacy compared to the real simulated vaccine efficacy ve = 90% for all four designs over the course of the trial.

When we estimated the bias in vaccine efficacy estimates at the end of the trial, we found that first-OSWCT underestimated vaccine efficacy by 3.0%, data-OSWCT overestimated vaccine efficacy by 1.4%, and peak-OSWCT overestimated vaccine efficacy by 0.7%. In contrast, SWCT underestimated vaccine efficacy by 0.87%. Fig 12 shows the change in bias over the course of the trial for all designs. We further investigated the bias of all designs when vaccine efficacy (*ve*) is set to zero (see S1 Appendix), on average the bias was 0.7% for SWCT, 0.1% for first-OSWCT, -0.7% for data-OSWCT, and -0.4% for peak-OSWCT. We estimated the corresponding type I error for SWCT to be 3.1%, for first-OSWCT 2.8%, for data-OSWCT 3.3%, and for peak-OSWCT to be 3.6%.

**Fig 13:**
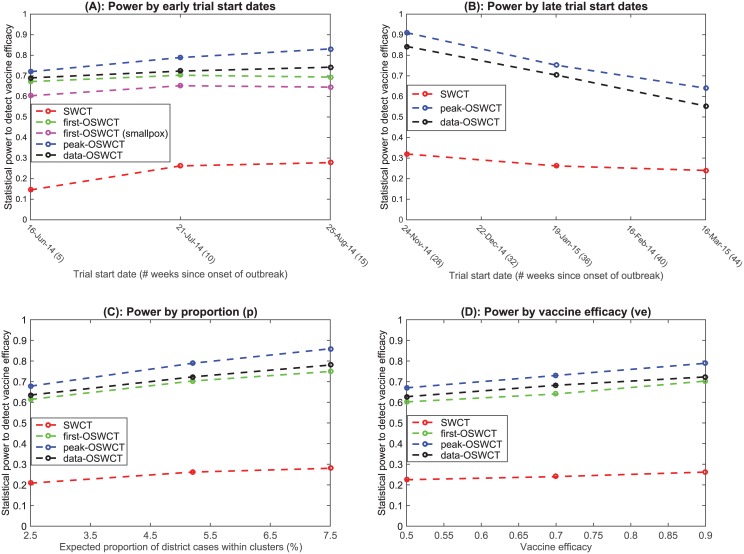
Statistical power for trial designs. (A) Power by early trial start dates. (B) Power by late trial start dates. (C) Power by proportion (p). (D) Power by vaccine efficacy (ve). SWCT: random ordering of clusters; data-OSWCT: clusters are ordered based on observed cases 2 weeks previously; first-OSWCT: clusters are ordered based on projected first case occurrence; peak-OSWCT: clusters are ordered based on the highest weekly projected incidence.

We found that the ordered study designs first-OSWCT, data-OSWCT, and peak-OSWCT had superior statistical power when compared to the standard SWCT design. In the baseline simulations, where we assumed the trial started 5 weeks after the onset of the outbreak, the ordered designs all had similar power to detect a vaccine efficacy of 90% with power ranging from 65% to 72%, compared to a power of 14% for the standard SWCT design. As we simulated later trial start dates, first-OSWCT design performed less efficiently compared to data-OSWCT and the peak-OSWCT designs (Fig 13A).

When we simulated trials with a start date after all clusters had observed their first case, we only simulated the SWCT, data-OSWCT, and peak-OSWCT designs, and no longer included the first-OSWCT design. The statistical power to detect a vaccine efficacy of 90% was 32% for SWCT, 84% for data-OSWCT, and 91% for peak-OSWCT (Fig 13B). While all the OSWCT designs were more efficient than the SWCT design regardless of when the trial started, all designs lost efficiency when the trial start date was delayed. Similarly for trial start date set at 10 weeks after onset of outbreak, Fig 13C and 13D show that peak-OSWCT outperforms data-OSWCT, first-OSWCT, and SWCT regardless of the proportion (*p*) of cases that occur in high-risk groups, or the vaccine efficacy (*ve*). In S1 Appendix we show similar results when the time from vaccination until vaccine-induced immunity (*di*) varies.

## Discussion

Here we demonstrate the superior efficiency of novel “ordered stepped-wedge cluster trial” (OSWCT) designs in detecting Ebola vaccine efficacy when compared to the standard stepped-wedge cluster trial (SWCT). To our knowledge, we are the first to propose ordering clusters by timing of expected outcomes to increase the efficiency of a stepped-wedge cluster trial. Among the three ordered designs we evaluated, we found that ordering based on the highest weekly projected incidence (peak-OSWCT) was more efficient than ordering based on projected first case occurrence (first-OSWCT) or on observed highest cases in the prior two weeks (data-OSWCT) regardless of when a trial begins during the course of an Ebola outbreak. However, we also found that for trials starting within 5 weeks of the onset of an Ebola outbreak, the magnitude of the statistical power of all three ordered designs was similar. The preferred trial design may be influenced by available epidemiologic data. Both model-based approaches (first-OSWCT and peak-OSWCT) require knowing where the outbreak began. However, unlike the data-OSWCT, neither model-based approach requires surveillance in the two weeks before the trial begins. The peak-OSWCT is more dependent than the first-OSWCT on the parameterization of the model; hence without accurate estimates of model parameters from *a priori* knowledge, fitting to initial data on the outbreak, or both, the first-OSWCT approach is more desirable. We also found that if vaccine trials are delayed beyond the first 15 weeks of an outbreak then peak-OSWCT may be the optimal choice. Our findings support the importance of epidemiologic surveillance to inform vaccine trial design.

Our transmission model is similar to other EVD models [11, 23–25] in that we distinguish transmission that occurs from live patients in the community versus that which occurs in ETUs versus transmission during funerals. However, our model distinguishes between EVD patients with dry versus wet symptoms. We have shown that our model accurately fits the observed data when it takes into account metrics of disease control interventions such as ETU bed availability. This illustrates the importance of recording not only the incidence of disease in outbreak settings, but also tracking when and to what degree interventions are implemented.

We stress that the efficacy of the first-OSWCT and peak-OSWCT depends on the accuracy with which the transmission model predicts the order of cases. We sought to address the question of model misspecification using the ordering derived from a simulation for infectious disease with different natural histories (e.g. smallpox, varicella, and measles). Using our spatially explicit transmission model, we showed that the projected order of first case occurrence in each district of SL was robust to different transmission scenarios for smallpox, varicella, and measles. Fig 13A shows that when we derived the ordering from a smallpox model, we lost power compared to when we used the Ebola transmission model, but the power remained higher than that of a typical SWCT. This finding suggests that when planning a stepped-wedge vaccine trial for a filovirus outbreak in a resource-limited setting, one can approximately predict the order of first case occurrence in different regions and implement a first-OSWCT design by using current EVD transmission parameters combined with geospatial data from the affected country, even if the transmission parameters of the outbreak in truth differ substantially from those observed for EVD. We did not specifically explore the choice of optimal value for N (the number of top clusters from which the cluster to vaccinate is randomly chosen), however the two extreme choices to avoid would be to set N as the total number of clusters (which will be equivalent to the typical SWCT) or set N to be 1 (in which case there will not be any control group to compare to). More generally, a high value for N loses the advantage of an OSWCT while a very low value for N would lose efficiency because of the small number of controls to which to compare. Therefore, we anticipate the relationship between N and power to be non-monotonic. The specific shape of this relationship probably depends on a number of factors, including the distribution of number of cases per cluster. This aspect of optimal OSWCT design could be the subject of further investigation.

Gravity models have been previously used to model human mobility in the context of various outbreaks. For instance, Ashleigh et al. [26] used a gravity model to describe the 2010 cholera epidemic in Haiti and to capture the ordering of first case occurrence among the departments of Haiti, Viboud et al. [27] used it to characterize seasonal influenza dynamics in the United States, and more recently it was used by Silva et al. [28] to capture EVD transmission dynamics within and between Guinea, Liberia, and SL. To our knowledge, our study is the first to link a gravity model to order a stepped-wedge cluster trial.

For our ordering of first case occurrence, we found that the metapopulation model predicted cases to occur in the Western Area Rural and Urban districts sooner than actually occurred. This may be because these two districts (which consist of Freetown and the surrounding area) are more densely populated than the other districts of SL and that the gravity model gives more weight to more populated regions. This weight may be disproportionately large compared to their actual influence. Other limitations of the gravity model are that it does not take into account other factors that may influence migration such as weather conditions and social networks, both of which are known to impact migration in sub-Saharan Africa [29]. An alternative to measure migration between regions, thus improving the accuracy of predicted order of first case occurrence, could be to use data that directly capture human mobility, such as mobile phone data. Buckee et al. [30] described the use of call data records (mobile phone calls or text messages) to infer mobile phone users’ travel. These data can be used to estimate human mobility between regions and its impact on disease transmission. However, we reiterate that even the relatively simple gravity model can capture the spatiotemporal trends of an outbreak well enough that ordering clusters accordingly in a stepped-wedge cluster trial substantially increases the statistical power of the trial.

When the vaccine being tested is expected to be efficacious and the risk of infection is predicted to differ between clusters at given time, the OSWCT design increases the probability that those at higher risk of infection will be vaccinated, and therefore we expect the OSWCT design to prevent more cases. In this way, the OSWCT is ethically advantageous, in addition to its statistical advantages. However, all types of SWCTs are ethically advantageous only to the extent the lags between implementing the intervention between clusters are due to genuine logistical constraints, not an intentional decision to increase the statistical power of the trial.

In this study we did not consider other clinical trial designs such as a randomized clinical trial or a ring vaccination cluster-randomized trial. Because we also assumed the vaccine trial to be conducted in a small subgroup of the population (healthcare workers), we did not consider the indirect effect (herd immunity) of the vaccination trial on the overall disease dynamics. Our main aim was to evaluate how ordering clusters when the risk of infection is heterogeneous among them may affect the statistical power of a SWCT design in settings where SWCT design may be desirable due to either ethical or logistical reasons. Our results support OSWCTs as more efficient designs than the standard SWCT.

## Supporting Information

S1 Appendix Novel ordered stepped-wedge cluster trial designs for detecting Ebola vaccine efficacy using a spatially structured mathematical model. (PDF)
