# Supplementary material for: Novel Ordered Stepped-Wedge Cluster Trial Designs for Detecting Ebola Vaccine Efficacy Using a Spatially Structured Mathematical Model
Source: PLoS Negl Trop Dis. 2016 Aug 10;10(8):e0004866. doi: 10.1371/journal.pntd.0004866 (PMC4979980; doi:10.1371/journal.pntd.0004866)
Supplement: S1 Appendix — (PDF) [file pntd.0004866.s001.pdf]

Novel ordered stepped-wedge cluster trial designs for detecting Ebola vaccine efficacy using a spatially-structured mathematical model

Ibrahim Diakite, Eric Q. Mooring, Gustavo E. Velásquez, and Megan B. Murray

Online supplementary Appendix

June 7, 2016

1 Model description and equations

1.1 Ebola transmission model

We constructed a simple SEIR model that captures Ebola virus disease (EVD) natural history, incorporating transmission scenarios in the community, treatment units and during burial ceremonies . Eight major compartments in the model depicted in figure 3 (main text) reflect the dynamics of the disease. The state parameters are described below, and superscripts 'c' and 'etu' indicate state that corresponds to a host living in the community or in an Ebola treatment unit respectively.

- S - Susceptible to infection by EVD;
- E - Infected by EVD but do not have any symptom nor infectious to others;
- F_e^c and F_e^{etu} - Infected by EVD and have early or "dry" symptoms (fever, myalgia, headache, presence of oropharyngeal lesions, nausea, abdominal pain, and rash) but not yet infectious to others;
- I^c and I^{etu} - Infected by EVD and present late or "wet" symptoms (vomiting, diarrhea, coughing, or hemorrhage from any site e.g. epistaxis, hematemesis, hematochezia, melena) and infectious to others;
- F_u - Dead bodies due to EVD and infectious to others during funeral practices;
- R - Recover from EVD;
- N - Total host population.

The full set of dynamic equations is given by the following set of ordinary differential equations, where key intervention measure's parameters are time depending, and Table 1 (main text) provides the full list of parameters with descriptions and values.

$$
\begin{aligned}
\frac{dS}{dt} &= -\left[\beta^c(t)I^c + \beta^{etu}(t)(I_1^{etu} + I_2^{etu}) + \beta^f(t)F_u\right] S/N,\\
\frac{dE}{dt} &= \left[\beta^c(t)I^c + \beta^{etu}(t)(I_1^{etu} + I_2^{etu}) + \beta^f(t)F_u\right] S/N - \alpha E,\\
\frac{dF_e^c}{dt} &= \alpha E - [\phi(1-\theta(t)) - \alpha_h\theta(t)]F_e^c,\\
\frac{dI^c}{dt} &= \phi[1-\theta(t)]F_e^c - [\alpha_h\theta(t) + r(1-\theta(t))(1-\delta_1(t))]I^c\\
&\quad - [k(t)\delta_1(t)(1-\theta(t))\gamma + (1-k(t))\delta_1(t)(1-\theta(t))\gamma]\, I^c,\\
\frac{dF_e^{etu}}{dt} &= \alpha_h\theta(t)F_e^c - \phi_h F_e^{etu},\\
\frac{dI_1^{etu}}{dt} &= \phi_h F_e^{etu} - [r(1-\delta_2(t)) + \gamma\delta_2(t)]\, I_1^{etu}\\
\frac{dI_2^{etu}}{dt} &= \alpha_h\theta(t)I^c - [\gamma_h\delta_2(t) + r_h(1-\delta_2(t))]\, I_2^{etu},\\
\frac{dF_u}{dt} &= (1-k(t))\delta_1(t)(1-\theta(t))\gamma I^c - \gamma_f F_u,\\
\frac{dR}{dt} &= r(1-\theta(t))(1-\delta_1(t))I^c + r(1-\delta_2(t))I_1^{etu} + r_h(1-\delta_2(t))I_2^{etu}.
\end{aligned}
$$

The above equations describe the transmission process inside a "patch" (a region). The parameters $\beta^c(t)$, $\beta^{etu}(t)$, and $\beta^f(t)$ are the transmission coefficients in the community, in Ebola treatment units and during funerals respectively. α^{-1} is the natural EVD incubation time, ϕ^{-1} is the time from onset of "dry" symptoms to onset of "wet" symptoms, r^{-1} is the duration of infectiousness for survivors (duration of "wet" symptoms), and γ^{-1} is the time from onset of "wet" symptoms to death. $\theta(t)$ is the probability of been admitted in ETU's upon any symptoms. α_h^{-1} is the time to hospitalization, ϕ_h^{-1} is the time to onset of "wet" symptoms for those hospitalized with "dry" symptoms, r_h^{-1} and γ_h^{-1} are respectively the time till recovery or death after hospitalization with "wet" symptoms, f^{-1} is the duration of funeral practices. $\delta_1(t)$ and $\delta_2(t)$ are the case fatality rates in the community and in ETU's respectively, and $k(t)$ is the probability of proper funerals when an Ebola patient die within the community. I_1^{etu} can be interpreted as the newly infected with "wet" symptoms in ETUs, and I_2^{etu} as those who enter the treatment units after been infected with "wet" symptoms for some time.

1.2 Gravity Model

Human mobility between the "patches" (regions) is described by the gravity model:

$$m(i,j) = \rho \frac{P(i).P(j)}{D(i,j)^n}, \quad i,j = 1,\ldots,T_r$$

where $m(i,j)$ is the mobility rate from regions i to region j, ρ is a scalar multiplied by the gravity matrix representing between-patch coupling, $P(i)$ is the population density of region i, $D(i,j)$ is the distance between 2 "patches", n is a power that determines the strength of the dependence of transmission rate on distance, and T_r is the total number of patches or regions. We assumed that individuals in the compartments (E), (F_e^c) and (I^c) may travel at rate m between the regions. Therefore the force of transmission is also a function of the migration rate and the following additional equations for transmission between "patches" (or regions) can be written:

$$\begin{aligned}
\frac{dS_i}{dt} &= -\beta_i^c(t)\sum_{j=1}^{T_r} m(i,j) I_j^c S_i/N_i, \\
\frac{dE_i}{dt} &= \beta_i^c(t)\sum_{j=1}^{T_r} m(i,j) I_j^c S_i/N_i + \sum_{j=1}^{T_r} m(i,j)E_j - \sum_{j=1}^{T_r} m(j,i)E_i, \\
\frac{dF_{ei}^c}{dt} &= \sum_{j=1}^{T_r} m(i,j)F_{ej}^c - \sum_{j=1}^{T_r} m(j,i)F_{ei}^c, \\
\frac{dI_i^c}{dt} &= \sum_{j=1}^{T_r} m(i,j)I_j^c - \sum_{j=1}^{T_r} m(j,i)I_i^c
\end{aligned}$$

where the subscript 'i' indicates the state or parameter value in the ith patch ($i = 1,\ldots,T_r$), and T_r represents the total number of regions considered.
The transmission model for EVD natural history coupled with a gravity model give the metapopulation model that captures the spatio-temporal spread of EVD where disease spreads within and between "patches" (regions).

2 Calibration and Parametrization

2.1 Initial Dynamics of Infection

We first calibrated the transmission model to capture the early disease dynamics in Sierra Leone (SL). We fitted the model to the cumulative cases count of EVD in SL from May 19 2014 to September 16, 2014 using the least square method to estimate $\beta^c(t)$, $\beta^{etu}(t)$, $\beta^f(t)$, and $\theta(t)$. We assumed that prior to the scale up of the interventions measures these parameters remained constant, and also during that time period when an Ebola patient within the community died we considered his probability $k(t)$ to receive proper funerals (that is the corpus is well handle without risk of infection to susceptible during funeral practices) to be zero.

2.2 Changing Dynamics of Infection over Time

To model the effects of intervention and behavioral changes on the disease's dynamic, we assumed that the transmission coefficients began to decrease exponentially after a more vigorous international response in September

2014 such that:

$$\begin{cases} \beta^c(t) = \beta_1^c + (\beta_0^c - \beta_1^c)e^{-q^c(t-\tau)} \\ \beta^{etu}(t) = \beta_1^{etu} + (\beta_0^{etu} - \beta_1^{etu})e^{-q^{etu}(t-\tau)} \\ \beta^f(t) = \beta_1^f + (\beta_0^f - \beta_1^f)e^{-q^c(t-\tau)} \end{cases}$$

where β_0^c, β_0^{etu}, and β_0^f are respectively the transmission coefficients in the community, in ETUs and during funerals before intervention date τ; q^c, q^{etu} and q^f are respectively the exponential decay rates from the initial transmissions to the final transmissions coefficients β_1^c, β_1^{etu}, and β_1^f. We refitted the transmission model to incorporate data from September 2014 to mid January 2015, and using the least square method we estimated q^c, q^{etu}, q^f, β_1^c, β_1^{etu} and β_1^f (Table 1 main text).

We found the case fatality ratio $\delta(t)$ in SL for the months of September through December 2014 to be best fitted to a cubic regression curve

$$\delta(t) = -1.543 \times 10^{-6}t^3 + 9.063 \times 10^{-4}t^2 - 0.173t + 11.407$$

We modeled the probability of hospitalization as a function of the cumulative number of beds in ETUs, the number of hospitalized patients, and the Ebola prevalence within the community. Given that beside the available beds in treatment units other factors such as distance to treatment units, and the infected individual's willingness to seek hospitalization are to be considered for the probability of hospitalization, when there were more available beds in treatment units than infected cases in the community, we corrected our value by the reported proportion of hospitalized cases as of June 2015.

$$\theta(t) = min\left\{\frac{\text{Total number of beds}(t) - [F^{etu}(t) + I_1^{etu}(t) + I_2^{etu}(t)]}{F_e^c(t) + I^c(t)}, 0.80\right\},$$

where, 0.80 is the reported proportion of hospitalized cases as of June 2015.[1]

Finally we parametrized the change in the proportion of safe burial $k(t)$ in the community after scale up of international response to the EVD outbreak based on WHO reported data on performance indicators. We obtained $k(t)$ to increase linearly in the period of September 2014 to January 2015, with $k(t) = 0.0136 * t - 1.827$.

3 Ordering and Timing of first case occurrence and model forcast of cases

We combined the equations for the Ebola transmission model and the gravity model, and for a range of values of ρ and n (the unknown parameters for the gravity model) we subsequently fitted the model to the WHO reported order and timing of first case occurence in each of the 14 districts of Sierra Leone. We evaluated the goodness of fit by calculating at each model fit the Spearman correlation coefficients between the model's projection and the observed data on order and timing of case occurence. Figure 10 in the main text shows a comparison of the model projection of the order and timing of first case occurrence at the district-level in Sierra Leone compared to the WHO reported data. We also used the model to project the order and timing of first cases occurrence at the chiefdoms level in Sierra Leone, we assumed that infection between districts ceased after the onset of international response in the country in late September 2014. Figure S1 below shows a color map of the spatiotemporal trend in infection cases at the chiefdoms-level in Sierra Leone.

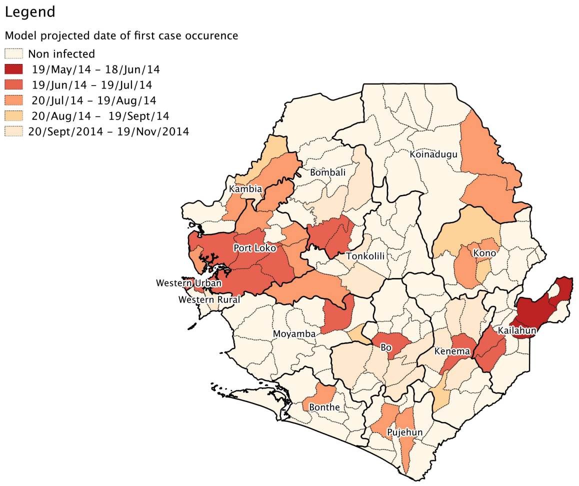

Figure S1: Spatial model's projection of order and timing of first case occurrence in the affected chiefdoms of Sierra Leone.

We finaly used the spatial model to forecast infectious cases at the districts-level, the best fit parameters (Table 1 in the main) for the Ebola natural history model obtained at the country level were used for each district in the metapopulation model and we rescaled the transmissions coefficients to qualitatively match the districts-level data. Figure S2 shows the model projection of the cumulative cases in some of the main affected districts of Sierra Leone.

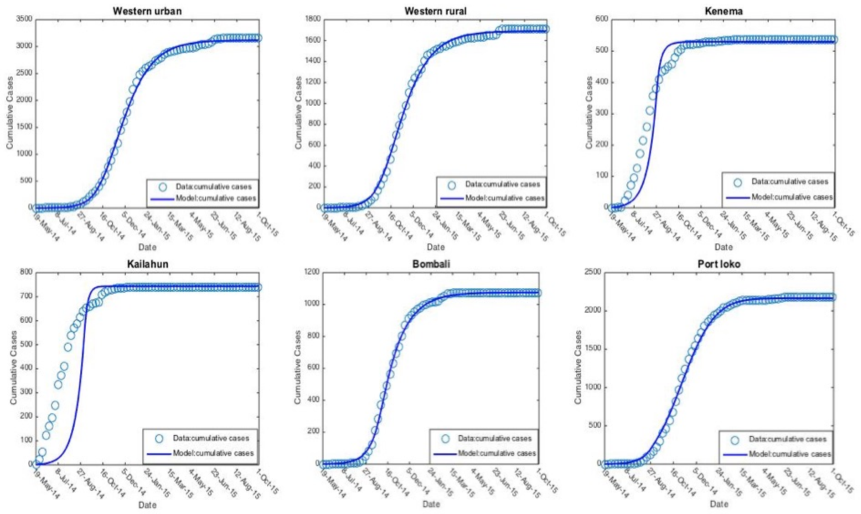

Figure S2: Cumulative cases in the districts of Sierra Leone.

4 Model Validation

To test the extent to which the spatial model's ordering of first cases depended on the disease's parameters used in the Ebola transmission model, we simulated a hypothetical outbreak of smallpox, chickenpox and measles in the same regions of SL. We considered a simple SEPIR model used by Burcu et al. [10], where susceptible individuals (S) when infected are moved into the exposed compartment (E) where they are not yet infectious to others, after an incubation period they become prodromal (P) with reduced infectivity. After certain days the prodromal show sign of first symptoms where they are moved to the infectious compartment (I) and they are assumed fully infectious to others, afterward they can either recover or die. We drawn the model's parameter values from published literature (Table S1). We then coupled the transmission model for each disease to the gravity model (with our initial estimate values for the gravity parameters ρ and n) and we reran the spatial model to obtain new ordering of first case occurrence. We measured the correlation between the spatial model's ordering of EVD outbreak versus smallpox, chickenpox, and measles outbreak by evaluating the Spearman correlation coefficients.

Table S1: Disease parameters

Parameters description	Smallpox Values	Chickenpox Values	Measles Values	References
Incubation period	12 days	14 days	11 days	[7, 8][a], [11][b], [12, 13][c]
Prodromal period	3 days	2 days	3 days	[7, 8][a], [11][b], [12, 13][c]
Infectious period	16 days	16 days	14 days	[7, 8][a], [11][b], [12, 13][c]
Case fatality rate	30%	3×10^{-14}	15%	[7, 10][a], [11][b], [12, 13][c]
Basic reproductive ratio of infectious cases	5	7	12	[9][a], [14][b], [9][c]
Reduced infectivity rate for prodromal cases	0.3	-	-	[10][a]
Isolation rate for prodromal cases	0.3	-	-	[10][a]
Hospitalization rate of infectious cases	0.08	-	-	[10][a]

(a), (b), and (c) are references for Smallpox, Chickenpox and Measles respectively. (-): values were assumed as for smallpox outbreak.

5 Vaccine Design Simulation and Calculation of Statistical Power

As explained in the main text, we simulated and assessed the statistical power of different type of vaccine trial designs: the standard stepped-wedge cluster trial (SWCT) and our proposed "ordered stepped-wedge cluster trial" (OSWCT). In the SWCT, we assumed that each week one unvaccinated cluster was randomly chosen from the control group to receive vaccination until all clusters have crossed over from the control to the treatment arm. Whereas, in the OSWCT, we based the ordering on either the observed incidence data, or the projected order of first case occurrence in each district, or based on the districts with projected highest incidence by our spatially structured Ebola mode. More specifically for instance for the peak-OSWCT (similar for first-oswct or data-OSWCT) at each time step of the trial we considered at most the four unvaccinated clusters that are predicted to have the highest incidence at that week, and among those four we randomly assigned one cluster to receive vaccination and the remaining 3 clusters served as control group. This step was repeated until all clusters crossed over from control to treatment group.

For all designs we assumed that the trial would be conducted in hight risk individuals such as healthcare workers and other front line workers, and that each simulated cluster had the same size, CS, or same number of individuals. To ensure that the clusters were geographically distinct with different infection risks, we chose one cluster to reside in each of the 14 districts of SL. To simulate the designs we followed Bellan et al [5], and we evaluated the cluster-level hazard by assuming that without effective vaccination a proportion p of the reported district cases would occur within the corresponding cluster. Thus we calculated the hazard in each cluster at each week of the trial as follow:

$$H_p(\text{week}) = \frac{(\text{total reported district cases in a week}) \times p}{CS \times 1 \text{ week}}$$

We assumed that individuals in the clusters became infected following an exponential distribution waiting time, and each week each individual hazard was calculated as $H_p(\text{week}) \times \varepsilon$, where ε captures the expected variation in individual risk and $\varepsilon \sim \ln \mathcal{N}(1, 1)$. We assumed that clusters that crossed over to the treatment arm were fully vaccinated within a week with a vaccine efficacy of ve, and that it takes a delay di to develop protective immunity after vaccination. Consequently we assumed that the risk of infection was reduced by $1 - ve$ for individuals in the vaccinated clusters that had passed the delay di.
Table S1 gives the values of the vaccine's parameters used in our baseline scenario, in sensitivity analyses we varied the trial start date, the proportion p, the vaccine efficacy ve, and the delay time until protective immunity di. For the trial start dates, we explored two scenarios, first when the trial begins early in the outbreak (before September 2014) before all districts were predicted to have their first case, and when the trial began later in the outbreak where districts have observed their first cases. For the later scenario, in the simulated trial we dropped the ordering design based on first case occurrence as all the districts would have already observed their first case.

Table S2: Vaccine parameters

Parameters	Description	Values	Reference
CS	Cluster size	430	[2]
p	expected proportion of district case to occur with clusters	5.2%	[3]
ve	Vaccine efficacy	90%	[4]
di	delay to develop protective immunity after vaccination	1 week	[4]

Bellan et al [5] have shown that when the simulated data for the SWCT is analyzed with a non-parametric based method (a permutation test) it preserved the targeted false-positive rate set at $\alpha = 0.05$. We consequently

analyzed our simulated data for all trial designs with a permutation test. From the simulated data we analyzed the regression model

$$log(E(Y_{it})) = \beta_{vac}X_{it} + \beta_{ct}CT_{it} + log(PY_{it}) + \beta_{time}t + C_i,$$

where Y_{it} is the number of cases observed in cluster i at time t, X_{it} is the vaccine status of cluster i at time t, CT_{it} indicates if a cluster i has contributed or not cluster time from $[t, t+1)$,and PY_{it} is all person-time in cluster i from time t to $t+1$ (except during the time to develop protective immunity), and the model includes the autoregressive (AR1) covariance structure in C_i for cluster-level variation. And the estimated vaccine efficacy was computed as $\hat{ve} = 1 - exp(\hat{\beta}_{vac})$
To test the null hypothesis that vaccines has no effect on the outcome of vaccinated individuals, we kept the outcome value Y_{it} for each cluster at each week and we randomly permuted 1000 times the time at which clusters received vaccination. We then re-analyzed the permuted data with the above regression model and for each permuted data we computed a corresponding $\hat{\beta}^p_{vac}$. To decide wether or not to reject the null hypothesis we calculated the Wald statistics[6]

$$W^p = \frac{\hat{\beta}^p_{vac} - \beta_{vac}}{se(\hat{\beta}^p_{vac})},$$

and we calculated the permutation p-value by obtaining the value that were more extreme than that of the original non-permuted data (2-side test).

We further assed the bias for each design by setting the vaccine efficacy $ve = 0$, and the figure bellow shows the distribution of the estimated vaccine efficacy for 1000 simulations of each design. We also calculated the corresponding type1error as shown in the main text.

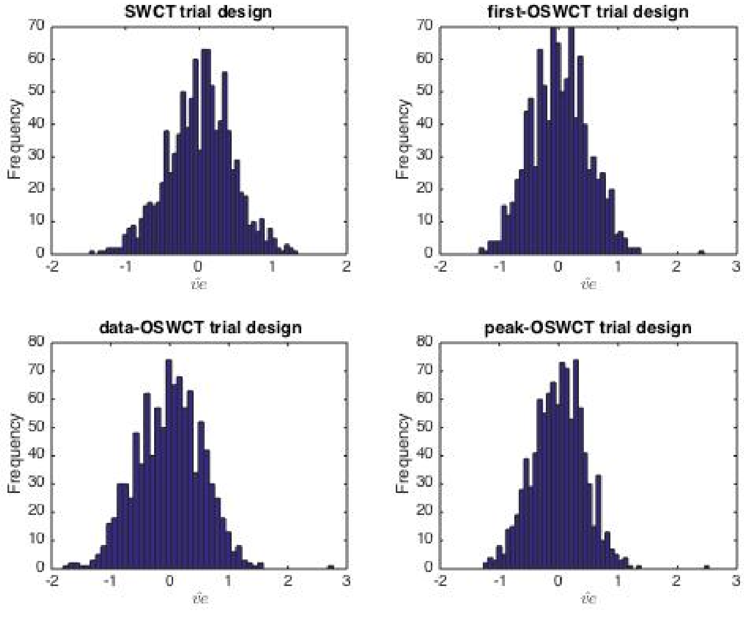

Figure S3: Distribution of the estimated vaccine efficacy, with true vaccine efficacy set to zero.

Similarly to the sensitivity analyses done in the main text for the effects of changes in trial start date, the expected proportion of district cases within clusters, and vaccine efficacy on the statistical power of each design; we also show here a corresponding analysis for changes in the time acquire immunity after vaccination. The vaccine efficacy was set to 90% with trial starting during the peak of the peak of the outbreak (weeks 28).

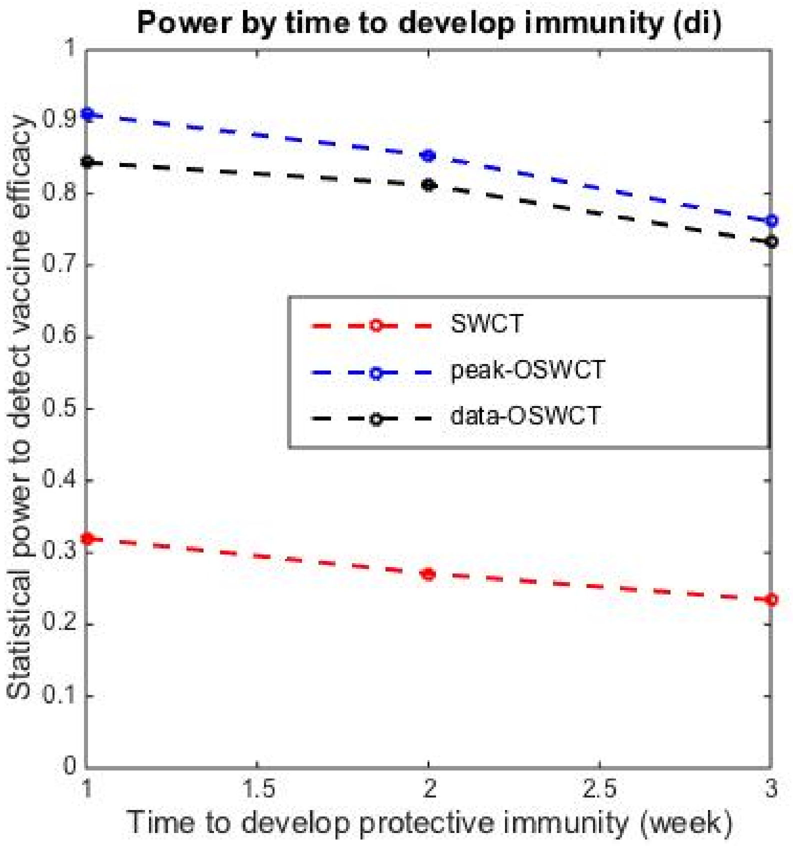

Figure S4: Power by the required time to develop immunity to EVD after vaccination. SWCT: random ordering of clusters; data-OSWCT: clusters are ordered based on observed cases 2 weeks previously; peak-OSWCT: clusters are ordered based on the highest weekly projected incidence.

References

[1] Ebola Situation Report : Ministry of Health and Sanitation -Sierra Leone. http://health.gov.sl/?page_id=583.

[2] Centers for Disease Control and Prevention.STRIVE (Sierra Leone Trial to Introduce a Vaccine Against Ebola). Available from https://clinicaltrials.gov/ct2/show/NCT02378753 NLM Identifier: NCT02378753.

[3] Ebola Virus Disease in Health Care Workers -Sierra Leone, 2014. http://www.cdc.gov/mmwr/preview/mmwrhtml/mm6349a6.htm. Accessed January 12, 2016

[4] Henao-Restrepo AM, Longini IM, Egger M, et al. *Efficacy and effectiveness of an rVSV-vectored vaccine expressing Ebola surface glycoprotein: interim results from the Guinea ring vaccination cluster-randomised trial.* Lancet. 2015;386(9996):857-866. doi:10.1016/S0140-6736(15)61117-5

[5] Bellan SE, Pulliam JRC, Pearson C a B, et al. *Statistical power and validity of Ebola vaccine trials in Sierra Leone: a simulation study of trial design and analysis.* Lancet Infect Dis. 2015;15(6):703-710. doi:10.1016/S1473-3099(15)70139-8

[6] Scott J. *Vaccine Efficacy Trials Using Stepped Wedge Design.* PhD dissertation, University of Washington. 2008. Available: http://library.harvard.edu

[7] F. Fenner, D.A. Henderson, L. Arita, Z. Jezek and L.D. Ladnyi, (1988) *Smallpox and Its Eradication. Publisher: World Health Organization, Geneva.*

[8] Longini, Ira M., et al.*Containing a large bioterrorist smallpox attack: a computer simulation approach.* International Journal of Infectious Diseases 11.2 (2007): 98-108.

[9] History and Epidemiology of Global Smallpox Eradication From the training course titled *Smallpox: Disease, Prevention, and Intervention.* The CDC and the World Health Organization. Slide 16-17. http://www.bt.cdc.gov/agent/smallpox/training/overview/pdf/eradicationhistory.pdf

[10] Adivar, Burcu, and Ebru Selin Selen. *Compartmental disease transmission models for smallpox.* Discrete and Continuous Dynamic Systems, American Institute of Mathematical Sciences, Springfield, MO, USA, Sept (2011): 13-21.

[11] Public Health Agency of Canada: Varicella (Chickenpox). http://www.phac-aspc.gc.ca/im/vpd-mev/varicella-eng.php

[12] Centers for Disease Control and Prevention: Measles (Rubeola). http://wwwnc.cdc.gov/travel/yellowbook/2016/infectious-diseases-related-to-travel/measles-rubeola

[13] World Health organization: Measles. http://www.who.int/mediacentre/factsheets/fs286/en/

[14] Anderson, Roy M., Robert M. May, and B. Anderson. *Infectious Diseases of Humans Dynamics and Control (Oxford Science Publications)*. New York: Oxford UP, USA, 1992.
